# I’ll Show You the Way: Risky Driver Behavior When “Following a Friend”

**DOI:** 10.3389/fpsyg.2017.00705

**Published:** 2017-05-09

**Authors:** Jaimie McNabb, Michael Kuzel, Rob Gray

**Affiliations:** ^1^Human Systems Engineering, Arizona State University Polytechnic campus, MesaAZ, USA; ^2^4M Safety, PhoenixAZ, USA

**Keywords:** driving, friend following, social influence on driving behavior

## Abstract

Previous research examining social influences on driving behavior has primarily focused on the effects of passengers and surrounding vehicles (e.g., speed contagion). Of current interest was the interaction between drivers that occurs in a “following a friend” scenario, i.e., the driver of one vehicle (the leader) knows how to get to the desired destination while the driver of a second vehicle (the follower) does not. Sixteen participants drove through a simulated city in a driving simulator under three conditions: (i) a baseline condition in which they could choose their own route, (ii) a navigation system condition in which they were given audible route instructions, and (iii) a “follow a friend” condition in which they required to follow a simulated vehicle. In the follow a friend condition, drivers engaged in significantly more risky behaviors (in comparison to the other conditions) such as making more erratic and higher speed turns and lane changes, maintaining overall higher speed, as well as maintaining a shorter time headway when following a lead vehicle. These effects suggest a relationship to time pressure caused by a fear of getting lost.

## Introduction

There are many factors that can influence a driver’s decision about whether or not to engage in risky behaviors on the road (Groeger, 2000), however, one that has received relatively little attention is the social influence of other vehicle’s. Previous research examining social factors in driving has primarily examined either the effects of passengers in the driver’s own vehicle (e.g., Conner et al., 2003) or normative influences (e.g., Elliott et al., 2005). Research exploring the direct social effects of the traffic environment has almost exclusively focused on contagion effects of speed (e.g., Connolly and Aberg, 1993). The goal of the present study was to examine a relatively common social interaction that occurs on the roadway, but, to our knowledge, has not been previously studied: “following a friend.”

In the “following a friend” situation, the driver of one vehicle (the leader) knows how to get to the desired destination while the driver of a second vehicle (the follower) does not. This unique situation creates the potential for several different social influences on driving behavior. First, previous research has shown that familiarity with a route can lead drivers to reduce their fixation duration on traffic signs and drive at a higher speed (Martens and Fox, 2007). The possibility of these risky driving behaviors by the leader could potentially result in two effects on the follower: contagion and time pressure.

Previous research examining contagion effects on driving speed have revealed a variety of different consequences. First, drivers tend to overestimate the speed of other vehicles on the roadway and worry that other drivers will perceive they are driving too slow (Åberg et al., 1997). Overall, the net result of these social comparisons is that drivers may feel unnecessary pressure to match the speed of their vehicle to that of the other vehicles on the roadway (Connolly and Aberg, 1993). Furthermore, using this social comparison to reduce driver’s speeds, studies have found that when given feedback about a high proportion of drivers on the roadway that were not speeding, the social pressure to keep pace was reduced (Van Houten and Nau, 1983). Similar contagion effects have also been observed for driving behavior at traffic lights (Palat and Delhomme, 2016). Specifically, the presence of other vehicles in a driving simulator that were programmed to run yellow lights increased participants’ risky behaviors while approaching, going through, and starting from a stop at traffic lights as compared to when they were the only vehicle on the road. These contagion effect findings have been explained in terms of theories of impression management and self-presentation in which it is proposed we attempt to control the ways in which others regard us (Schlenker and Pontari, 2000).

Another factor that has been shown to increase risky behaviors is time pressure. As indicated by research findings, time pressure can lead to an underestimation of one’s own driving speed (Cœugnet et al., 2013), increased incidence of speeding (Åberg et al., 1997), a shortening of acceptable gaps for across path turns (Mahmassani and Sheffi, 1981), and an increased propensity to run red lights (Palat and Delhomme, 2016). Time pressure on the road has also been shown to increase a driver’s stress level, in particular, when there is time uncertainty involved (Cœugnet et al., 2013).

In sum, based on previous research, the “following the friend” driving scenario presents multiple ways that the follower driver could be induced into riskier driving behavior as a result of the social interaction with the leader. The goal of the present study was to specifically focus on the potential time pressure effects in this situation by removing possible contagion effects. To achieve this end, drivers were asked to follow a simulated “friend” vehicle that obeyed all traffic laws. During the drive, the participant (but not the simulated friend vehicle) faced a series of critical events that had the potential to induce risky driver behavior (e.g., a light changing yellow). In a within-subjects design, this “follow a friend” condition was compared to a condition in which drivers were asked to follow audible directions from a simulated global positioning system (GPS) navigation system and a baseline condition in which they could follow a route of their own choice. The experiment was designed to test the following predictions:

(i) During critical events, participants would engage in more risky driving behaviors in the follow a friend condition (as compared to the navigation condition) as evidenced by significantly higher speeds and a greater proportion of risky decisions (e.g., turning in front of an oncoming car or going through a yellow light).
(ii) Overall, participants would engage in more risky driving behaviors in the follow a friend condition (as compared to both the navigation and baseline conditions) as evidenced by significantly higher driver speeds during straight road segments, significantly shorter time headways when following a lead vehicle, and significantly faster lane changes.

## Materials and Methods

### Participants

Sixteen undergraduates from Arizona State University participated for partial fulfillment of an introductory psychology research requirement. All were native English speakers with normal or corrected-to-normal vision with a valid driver’s license. Participants ranged in age from 18 to 22 years (*M* = 21.2, *SD* = 1.8). This study was carried out in accordance with the recommendations of the Arizona State University Institutional Review Board with written informed consent from all subjects. All subjects gave written informed consent in accordance with the Declaration of Helsinki. The protocol was approved by the Arizona State University Institutional Review Board.

### Apparatus

**FIGURE 1:**
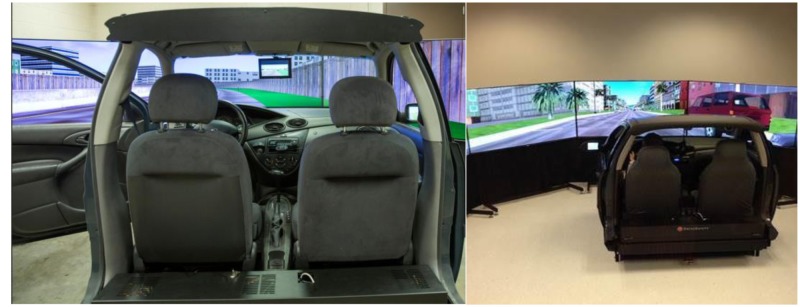
**The driving simulator**.

The DS-600c Advanced Research Simulator by DriveSafety^TM^ (shown in **Figure 1**) was used for the testing. This simulator was comprised of a 300° wraparound display, a full-width automobile cab (a Ford Focus) and a motion platform. The motion platform provided coordinated inertial cues for the onset of longitudinal acceleration and deceleration. The data recording rate was 60 Hz.

### Procedure

Participants were asked to drive in a simulated city and obey all traffic laws (e.g., posted speed limit, red lights, etc.). The posted speed limit was 35 mph and intermittent traffic was included. Drivers were given a 5-min baseline condition to become familiar with the driving simulator before proceeding to the baseline condition and the two route following conditions. In the baseline condition, drivers were asked to drive around the simulated city by taking any route that they wished.

There were two different route following conditions that were completed by all participants. In the navigation system condition, participants were asked to use audible directions, from a simulated GPS navigation system, in order to arrive at their final destination. Specifically, directions such as “Turn right on Acorn Street” would be given about 15 s prior to arriving at the critical intersection. The specific instructions given to participants in this condition were as follows:

“Please drive around the simulated city following the turn instructions given by the navigation system. For example, you might hear the system say “at the next intersection turn left at Park Street. Be sure to follow all speed limits and other regulation signs. Any questions?”

In the “following a friend” condition, participants were asked to drive around the same city while following the lead car in front of them to reach their final destination. The “simulated friend” car was programmed to drive in a specific route that was matched to the navigation condition. The lead vehicle always started at the same distance (12 m or roughly two car lengths) ahead of the follower, and was programmed to drive between 33 and 38 mph (with an average speed of 35 mph). The specific instructions given to participants in this condition were as follows:

“Please imagine you’re a in a situation where the blue car now sitting in front of you is driven by a friend of yours that knows how to get to your destination and has said to “follow me.” Be sure to follow all speed limits and other regulation signs. Any questions?”

During both of the route following conditions, three critical safety events were programmed to occur. First, upon approaching the initial critical intersection where the participant was required to make a right turn, a pedestrian entered the crosswalk from the left side of the road. Second, at the signaled intersection where the participant was required to make a right turn, a simulated vehicle was programmed to enter the intersection forcing the participant to choose whether to proceed with turn or wait for the oncoming vehicle to clear the intersection. Finally, at a signaled intersection where the participant was to proceed straight through, the traffic light was programmed to turn from green to yellow when the participant was exactly 2 s from the start of the intersection.

Each condition required approximately 8–10 min to complete. Similar to our previous studies (e.g., Scott and Gray, 2008) drivers always initially performed the baseline driving condition. The order of the two route following tasks was counterbalanced across participants. After the baseline driving condition participants were next given instructions about the two tasks. For all tasks, participants were asked to complete NASA-TLX questionnaire (which assessing workload, frustration, and demand) after the drive was complete. Participants received a 5-min rest between conditions to minimize simulator sickness and fatigue.

### Data Analysis

To assess risky driving behavior, the following variables associated with the three critical events were analyzed. For the pedestrian crossing event, we calculated the proportion of drivers that waited for the pedestrian to cross before making their turn, the temporal safety margin with pedestrian at turn onset and the mean speed and the standard deviation of steering wheel angle during the turn. For the left turn event, we calculated the proportion of drivers that waited for oncoming vehicle to go through the intersection before making their turn, the temporal safety margin at turn onset and the mean speed and the standard deviation of steering wheel angle during the turn. Finally, for the yellow light event, we calculated the proportion of drivers that stopped at the intersection and the maximum longitudinal acceleration through the intersection. Proportions were compared using the *z*-score test of proportions while all other variables were analyzed using pairwise *t*-tests.

In addition to these critical event variables, we also collected and analyzed some general variables for all three driving conditions. These were mean driving speed (averaged over all straight road sections), mean time headway (averaged across all situations in which the participant was within 5 s of another simulated vehicle) and the mean maximum lateral acceleration (averaged across all lane changes). These variables were analyzed using one-way repeated measures analysis of variance (ANOVAs).

The NASA-TLX task data were analyzed using a repeated measures multivariate analysis of variance (MANOVA).

For all results reported, statistical significance is set at *p* < 0.05. Effect sizes were calculated using partial eta squared ($\eta_{p}^{2}$) for ANOVAs and Cohen’s *d* for *t*-tests for all significant findings.

## Results

### Pedestrian Crossing Event

**FIGURE 2:**
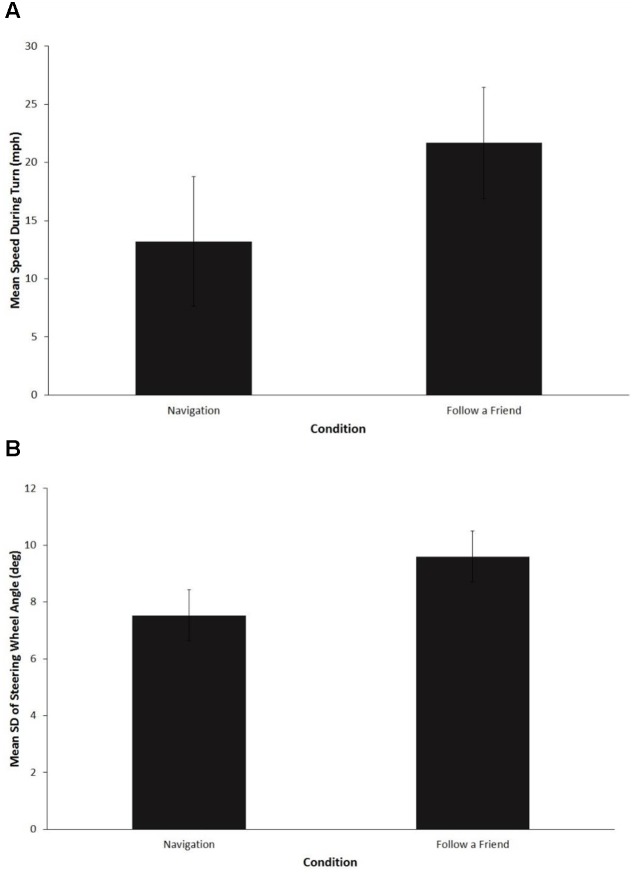
**Driving behavior for the pedestrian crossing event.** **(A)** Mean driving speeds (mph). **(B)** Mean standard deviation of steering wheel angle (degrees). Error bars are standard deviations.

Overall, the proportion of drivers that yielded to the pedestrian at the right-hand turn was calculated. The results indicate that significantly more drivers stopped and allowed the participant to cross the intersection in the navigation condition (44%) as compared to the “follow a friend” condition (0%), *z*(30) = 3.42, *p* = 0.002. **Figures 2A,B** show, respectively, the mean speed and mean standard deviation of steering wheel angle during the execution of the right turn at the pedestrian crossing intersection. A paired samples *t*-test revealed that mean driving speed was significantly higher in the “follow a friend” condition, *M* = 21.7 (*SD* = 4.8), as compared to the navigation condition, *M* = 13.2 (*SD* = 5.6), *t*(15) = 5.5, *p* < 0.001, *d* = 1.4, 95% CI 0.67–2.1. Mean variability of steering wheel angle was also significantly greater in the “follow a friend” condition, *M* = 9.6 (*SD* = 1.0), as compared to the navigation condition, *M* = 7.5 (*SD* = 0.9), *t*(15) = 7.5, *p* < 0.001, *d* = 1.9, 95% CI 1.0–2.7.

### Left-Turn with Oncoming Vehicle Event

**FIGURE 3:**
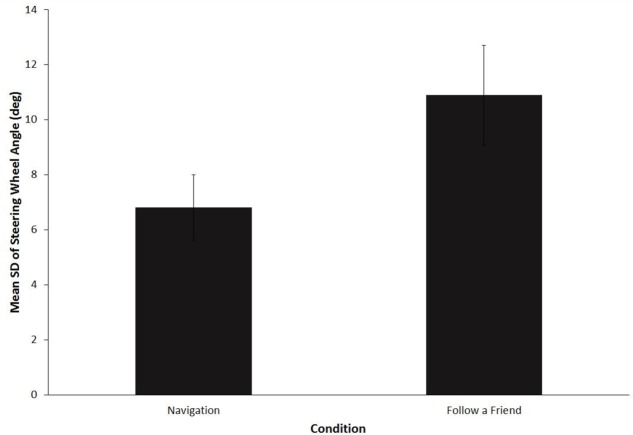
**Mean standard deviation of steering wheel angle (degrees) for the left turn critical event.** Error bars are standard deviations.

All drivers chose to turn in front of the oncoming vehicle in both conditions. **Figure 3** shows the mean standard deviation of steering wheel angle during the execution of the left turn. A paired samples *t*-test revealed that the standard deviation was significantly greater in the “follow a friend” condition, *M* = 10.9 (*SD* = 1.8), as compared to the navigation condition, *M* = 6.7 (*SD* = 1.1), *t*(15) = 7.2, *p* < 0.001, *d* = 1.8, 95% CI 0.98–2.6. There was no significant difference between the mean speed during execution of the turn for the navigation (*M* = 18.4, *SD* = 6.7) and “follow a friend” (*M* = 18.7, *SD* = 3.7), *p* = 0.9, *d* = 0.05, 95% CI -0.44 to 0.53.

### Yellow Light Event

**FIGURE 4:**
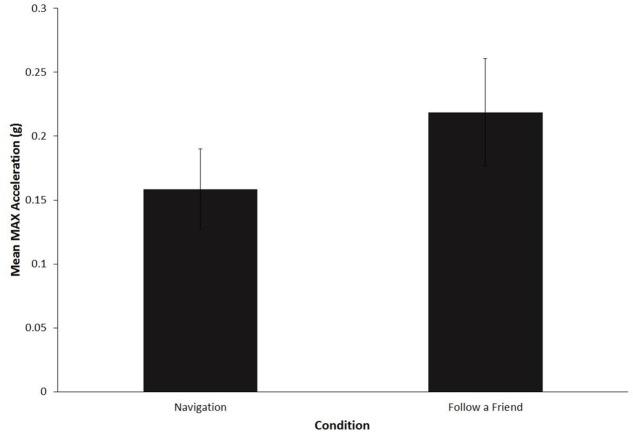
**Mean maximum longitudinal acceleration (*g*) for yellow light critical event.** Error bars are standard deviations.

All drivers in both route follow conditions choose to go through the yellow light rather than stopping at the intersection. **Figure 4** shows the mean maximum longitudinal acceleration when going through the intersection. A paired samples *t*-test revealed that the maximum acceleration was significantly greater in the “follow a friend” condition, *M* = 0.2 (*SD* = 0.04), as compared to the navigation condition, *M* = 0.15 (*SD* = 0.03), *t*(14) = 4.5, *p* < 0.001, *d* = 1.2, 95% CI 0.49–1.8.

### General Driving Behavior

**Table 1** shows the values for the different driving variables compared for the three different conditions. One-way repeated measures ANOVAs performed on these data revealed significant differences in mean driving speed, *F*(2,30) = 13.7, *p* < 0.001, $\eta_{p}^{2}$ = 0.48, 95% CI 0.18–0.63, mean time headway, *F*(2,28) = 15.6, *p* < 0.001, $\eta_{p}^{2}$ = 0.53, 95% CI 0.22–0.67, and mean time to complete a lane change maneuver, *F*(2,28) = 7.8, *p* = 0.02, $\eta_{p}^{2}$ = 0.36, 95% CI 0.07–0.54. In all cases, this was due to the fact that drivers in the follow a friend conditions engaged in riskier behaviors as compared to the other two groups.

**Table 1:** Means and standard deviations for general driving behavior measures.

Variable	Baseline	Navigation	Follow a friend
Speed (m/s)	35.1 (3.9)	33.9 (4.1)	39.2 (3.6)
Time headway (s)	2.5 (0.43)	2.8 (0.92)	1.6 (0.35)
Lane change (s)	3.9 (0.78)	4.8 (1.3)	2.9 (0.89)


### NASA-TLX

The MANOVA performed on the NASA-TLX task data revealed a significant main effects of driving condition, *F*(12, 50) = 2.98, Wilks λ = 0.30, *p* = 0.003, $\eta_{p}^{2}$ = 0.41, 95% CI 0.07–0.47. **Table 2** shows the means for mental, physical, and temporal demand, as well as task performance, required effort and overall frustration dimensions of the NASA-TLX questionnaire. The ANOVAs performed on each of the items revealed significant effects of condition on mental demand, *F*(2, 30) = 6.5, *p* = 0.005, $\eta_{p}^{2}$ = 0.30, 95% CI 0.04–0.49, physical demand, *F*(2,30) = 5.4, *p* = 0.01, $\eta_{p}^{2}$ = 0.26, 95% CI 0.02–0.46, and temporal demand, *F*(2,30) = 16.8, *p* < 0.001, $\eta_{p}^{2}$ = 0.52, 95% CI 0.24–0.67. For each of these variables, the highest mean score occurred in the follow a friend condition.

**Table 2:** Mean task-load response ratings captured from the NASA-TLX form.

	NASA-TLX measures				
	
Condition	Mental demand	Physical demand	Temporal demand	Performance	Effort	Frustration
Baseline	6.9 (5.4)	4.6 (3.7)	4.6 (3.0)	6.4 (4.6)	7.8 (5.5)	7.8 (6.3)
Navigation	9.5 (5.2)	6.1 (4.2)	6.2 (4.2)	8.3 (4.5)	9.0 (5.7)	8.3 (6.0)
Follow a friend	10.3 (5.5)	6.8 (5.0)	10.3 (5.9)	7.3 (5.1)	9.3 (4.9)	9.6 (6.5)


## Discussion

The aim of the present study was to investigate risky driving behaviors associated with the “following a friend” scenario. To achieve this end, a simulation of this situation was compared with a baseline condition, in which drivers could choose their own route, and a condition that required drivers to follow auditory directions given by a navigation system. We hypothesized that the drivers asked to “follow a friend” would engage in more risky behaviors both for the critical pre-programmed events in the simulation (pedestrian crossing, left turn in front of oncoming vehicle and yellow light at intersection) and in their general driving behavior. For the most part, these predictions were supported. In terms of the critical events, in the follow a friend condition drivers made turns that were higher speed and more erratic (i.e., high steering angle variability) and had a higher maximum acceleration when driving through intersections as compared to behavior in the navigation condition. For instance, in the pedestrian crossing critical event, a significantly higher proportion of drivers made the riskier choice (cutting in front of the pedestrian) as compared to waiting for the pedestrian to complete the crossing. We propose that similar differences in decision making did not occur for the other two critical events in our study because the temporal margins were not small enough. In terms of general driving performance, drivers in the follow a friend condition drove significantly faster, had significantly shorter time headways when following other vehicles, and quicker lane changes as compared to drivers in both the navigation and baseline conditions.

One notable attribute of the simulation was that in all three conditions the ambient traffic obeyed the traffic laws and (on average) drove at the simulated speed limit. This was true even for the leader vehicle in the follow the friend condition. Even though this vehicle’s speed varied, the average was 35 mph. Furthermore, at each of the critical events the lead car was not exposed to the potential for risky behaviors, i.e., there was no pedestrian in the instruction, there was no oncoming vehicle at left turn, and the traffic signal remained green at the critical intersection. Therefore, the risky driving behaviors found in the follow a friend condition were not due to social contagion type effects (e.g., Åberg et al., 1997), since the drivers in our study did not observe others driving in a risky manner.

A more likely explanation for the effects observed in the present study would seem to be time pressure. In a recent study by Rendon-Velez et al. (2016), many of the same effects were observed when drivers were given a time constraint to complete a course including higher driver speeds and more variability when making maneuvers. Consistent with this idea, in the present study, drivers’ had significantly higher ratings of temporal demand in the follow a friend condition as compared to the other conditions. However, in most previous research on time pressure in driving the pressure was created by an explicit external stressor (e.g., a countdown clock showing the time remaining to complete the course or a passenger urging the driver to go faster). As modeled by Wickens et al. (2004), these external time stressors serve to have direct influence on the information processing demands of the driver. In the present study, no such external time pressures were placed on the driver.

We propose that the risky driving behaviors in the follow a friend scenario are most likely the results an unique type of time pressure created by the fear of getting lost. In a study examining fears in driving, Taylor et al. (2000) found that “getting lost” was a common fear amongst drivers, ranking in the top 10 of driving-related fears. During a typical following a friend scenario there are multiple opportunities for the following vehicle to fall behind the lead vehicle. In such cases, the follower is faced with the possibility of not knowing where they are going. The present study attempted to recreate these types of situations. The fear of losing contact with the leading vehicle and getting lost is the most likely explanation for why drivers in the present study were more likely to cut in front a pedestrian to make a turn, maintain a shorter time headway, and execute turns at higher speeds and with higher accelerations. However, the risky behaviors were also observed in situations where participants were not likely to lose contact with the leader, e.g., when on a portion of straight road.

It is important to consider whether the “follow a friend” condition used in the present study was a valid representation of the analogous situation in real driving. First, is the possible that, in the simulation, drivers placed more weight on the goal of keeping up with the lead vehicle (as opposed to the goal of driving safely) than they would in real driving resulting in an overestimation of risking driving behaviors in the present study. We argue that this what not the case because there is previous research both from simulator tests and observations of real driving showing that drivers are readily willing to trade off safety for factors such as convenience, time, and excitement (e.g., Jackson and Blackman, 1994; Trimpop, 1996). Furthermore, it is likely the reward for successfully following the lead vehicle to the destination is higher in real driving as the driver presumably has some motivation for arriving at the location (e.g., a party) as opposed to the completely meaningless destination used the present study. In future research this could be addressed by introducing penalties for unsafe driving in the simulation (e.g., speeding tickets). A second issue concerns the instructions given to the participant in the “follow a friend” condition. In designing these, our goal was to recreate the type of communication that would naturally occur between two drivers (e.g., the phrase “follow me”). However, it is possible that this language may have led to participants treating the condition as an imitation task, i.e., their goal was to copy what the lead vehicle was doing. We would argue that this was unlikely to have occurred because the lead vehicle did not actually engage in any risky driving behaviors in the “follow a friend” condition and, therefore, if drivers had perfectly imitated its behavior there would have been null findings. Nevertheless, it will be important for future research to examine the how the risky driving behaviors observed in this situation vary as a function of the instructions given by the lead driver.

There are some important practical applications of the present findings. First, given the increase in risky driving behaviors, drivers would benefit by avoiding involvement in following friend scenarios. A better solution would be for the leader to provide the follower the route, e.g., via a map on a navigation system or a smart phone. Nevertheless, if direct following is necessary, it is critical that the lead vehicle reduce the likelihood of losing contact with the following vehicle by reducing their speed and making decisions that account for the driver following them (i.e., waiting until there is a large gap to make a left turn, waiting to change lanes until they both can safely change lanes). Most concerning, and of further research interest are situations in which teenagers are following one another to reach a destination, such as a party. In this specific situation when the lead driver is disobeying the rules of the road, such as driving at increased speeds and swerving through traffic erratically, the following driver may not only feel the pressure to not lose them, but more importantly, justify mimicking these same behaviors in order to stay together thus causing potential for serious driving incidents.

## Author Contributions

All authors listed, have made substantial, direct and intellectual contribution to the work, and approved it for publication.

## Conflict of Interest Statement

MK is affiliated with a commercial company, 4M Safety. The other authors declare that the research was conducted in the absence of any commercial or financial relationships that could be construed as a potential conflict of interest.
